# Results of the Second Phase of the GER-e-TEC Experiment concerning the Telemonitoring of Elderly Patients Affected by COVID-19 Disease to Detect the Exacerbation of Geriatric Syndromes

**DOI:** 10.3390/jpm11111117

**Published:** 2021-10-29

**Authors:** Abrar-Ahmad Zulfiqar, Delwende Noaga Damien Massimbo, Mohamed Hajjam, Bernard Geny, Samy Talha, Jawad Hajjam, Sylvie Erve, Amir Hajjam, Emmanuel Andrès

**Affiliations:** 1 Service de Médecine Interne, Diabète et Maladies Métaboliques de la Clinique Médicale B, Hôpitaux Universitaires de Strasbourg, Equipe EA 3072 “Mitochondrie, Stress Oxydant et Protection Musculaire”, Faculté de Médecine, Université de Strasbourg, 67000 Strasbourg, France; emmanuel.andres@chru-strasbourg.fr; 2 Predimed Technology Society, 67300 Schiltigheim, France; delwende_noaga.massimbo@edu.univ-fcomte.fr (D.N.D.M.); mohamed.hajjam@predimed-technology.com (M.H.); 3 Service de Physiologie et d’Explorations Fonctionnelles, Hôpitaux Universitaires de Strasbourg, Equipe EA 3072 “Mitochondrie, Stress Oxydant et Protection Musculaire”, Faculté de Médecine, Université de Strasbourg, 67000 Strasbourg, France; bernard.geny@chru-strasbourg.fr (B.G.); samy.talha@chru-strasbourg.fr (S.T.); 4 Centre d’Expertise des TIC pour l’Autonomie (CenTich), Mutualité Française Anjou-Mayenne (MFAM)-Angers, 49000 Angers, France; jawad.hajjam@centich.fr (J.H.); sylvie.erve@centich.fr (S.E.); 5 Laboratoire IRTES-SeT, Université de Technologie de Belfort-Montbéliard (UTBM), Belfort-Montbéliard, 90000 Belfort, France; amir.hajjam@utbm.fr

**Keywords:** geriatric syndromes, COVID-19, MyPredi platform, alerts, telemonitoring

## Abstract

Background: Coronavirus disease 2019 (COVID-19) has wreaked health and economic damage globally. This pandemic has created a difficult challenge for global public health. The coronavirus disease 2019 (COVID-19) pandemic has necessitated the use of new technologies and new processes to care for hospitalized patients, including elderly patients. Our team developed a telemonitoring program focused on the prevention of geriatric syndromes, the “GER-e-TEC COVID study”. Methods: This second phase took place during the 3rd wave of the epidemic in France, between 14 December 2020 and 25 February 2021, conducted in the University Hospital of Strasbourg. Results: 30 elderly patients affected by COVID-19 disease were monitored remotely; the mean age was 85.9 years and a male/female ratio of 1.5 to 1.11 (36.7%) died during the experiment. The patients used the telemedicine solution for an average of 27.3 days. 140,260 measurements were taken while monitoring the geriatric syndromes of the entire patient group. 4675 measurements were recorded per patient for geriatric disorders and risks. 319 measurements were recorded per patient per day. The telemedicine solution emitted a total of 1245 alerts while monitoring the geriatric syndromes of the entire patient group. In terms of sensitivity, the results were 100% for all geriatric risks and extremely satisfactory in terms of positive and negative predictive values. Survival analyses showed that gender played no role in the length of the hospital stay, regardless of the reason for the hospitalization (decompensated heart failure (*p* = 0.45), deterioration of general condition (*p* = 0.12), but significant for death (*p* = 0.028)). The analyses revealed that the length of the hospital stay was not affected by the number of alerts. The results concerning the predictive nature of alerts are satisfactory. Conclusions: The MyPredi™ telemedicine system allows for the generation of automatic, non-intrusive alerts when the health of a COVID-19 elderly patient deteriorates due to risks associated with geriatric syndromes.

## 1. Introduction

An outbreak of pneumonia linked to a new coronavirus termed severe acute respiratory syndrome coronavirus 2 (SARS-CoV-2) was first reported in Wuhan, China, in December 2019 [1]. Coronavirus disease 2019 (COVID-19), which is caused by this virus, then rapidly spread globally, resulting in a pandemic. In France, The Alsace region in the northeast harboured an important COVID-19 cluster [2]. The clinical spectrum of COVID-19 ranges from the absence of symptoms to life-threatening severe acute respiratory distress syndrome (ARDS) and death, making the detection and isolation of COVID-19 cases complex and facilitating the spread of the virus [3,4].

Coronavirus disease 2019 (COVID-19) has wreaked health and economic damage globally. This pandemic has created a difficult challenge for global public health [5]. The coronavirus disease 2019 (COVID-19) pandemic has necessitated the use of new technologies and new processes to care for hospitalized patients, including elderly patients. Moreover, optimal management of chronic diseases (like heart failure, diabetes, cognitive disorders, chronic obstructive pulmonary disorders (COPD), cancer, chronic kidney diseases, etc.) in elderly during this pandemic time is a real challenge for health professionals. In our opinion, innovative technologies based on artificial intelligence (i.e., machine learning, big data) are going to build the future of chronic disease, and they invent the medicine of tomorrow.

Telemedicine refers to health care provision through information technologies and telecommunication systems [6]. Telemedicine is particularly important in the field of geriatrics, especially when it comes to the monitoring of elderly patients with chronic diseases. The development and implementation of a telemonitoring care pathway in the clinical workflow is challenging. In fact, the emergence of these technologies in the daily lives of these patients suffering from chronic disease has led to an improvement of the quality of life for patients. Nevertheless, the magnitude of its effects remains to date debatable or to be determined, especially with the variation in patients’ characteristics and methods of experimentation and in terms of medical and economic objectives.

Very few studies have focused on telemonitoring of the geriatric population affected by COVID-19 infection. The novelty of COVID-19 and the need for rapid action pose additional difficulties. Based on our extensive experiences with telemonitoring for patients with chronic diseases, we developed and implemented a telemonitoring care pathway for patients with COVID-19.

In fact, our team developed a remote monitoring platform designed to help prevent the deterioration of geriatric syndromes: the GER-e-TEC project [7,8].

With the MyPredi™ remote monitoring platform being used for the GER-e-TEC project, patients benefited from personalized and preventive care that improves their quality of life. This includes multidimensional care and the monitoring of several indicators that are not addressed by other projects, such as the risk of constipation, dehydration, iatrogenesis, pain, and sleep disorders [8].

As part of the “Ger-e-Tec” remote monitoring project, we conducted an initial experiment between 24 September 2019 and 24 November 2019. For these two months, the MyPredi™ platform was used on patients being monitored in an internal medicine unit at the University Hospital of Strasbourg (CHRU). This first phase, which involved 36 elderly subjects, produced convincing results and was published in the Journal of Clinical Medicine [8].

A second phase of telemonitoring using the MyPredi™ telemedicine solution will concern the study of elderly patients suffering from infection linked to COVID-19, in connection with geriatric risks. The main goal of the second phase of the GER-e-TEC study is to confirm the technological choices made during the first experimental phase [8] to compare the two groups which were monitored remotely using the MyPredi™ solution, and will allow us to evaluate the impact of COVID-19-related infections on the assessed geriatric risks.

## 2. Patients and Method

### 2.1. Objective

The main goal of our experiment was to evaluate the functioning and to test the ergonomics of our remote monitoring solution here with the MyPredi™ platform [5] on elderly patients suffering from COVID-19 infection, in order to prevent a decompensation of geriatric risk.

COVID-19 is characterized by a rapid change in the elderly patient’s condition, with major changes occurring over a few days. We aimed to develop and evaluate a system for monitoring patients with COVID-19.

### 2.2. Patients

The experiment took place during the 3rd wave of the epidemic, in France, in a unit dedicated to the care of elderly patients with COVID-19 infection, between 14 December 2020 and 25 February 2021, at the University Hospital of Strasbourg. All patients older than 65 years with COVID-19 infection were included.

All patients with confirmed COVID-19 identified by positive results on reverse transcription polymerase chain reaction (RT-PCR) of nasopharyngeal swabs were included (SARS-CoV-2–Gene RdRp).

Patients with COVID-19-related infection who were monitored by the telemedicine solution for less than 48 h were excluded. The exclusion criteria are identical to the first experimental phase.

### 2.3. Study Outline

During the experiment, the patients recorded their vital signs every day with the help of smart devices. This data was then sent directly to the intelligent platform to be processed and analyzed in the unit. The platform uses an algorithm to anticipate geriatric risk situations for elderly patients affected by COVID-19 disease.

During the experiment, the alerts were compiled in the order they were received. They were analyzed with regard to the clinical context at the time they were emitted, using the discharge letter and computer files (medical and nursing) of the patient in question. This analysis was performed retrospectively by two professionals involved in the present study in the unit but not in contact with the caregivers who cared for the patients on a daily basis. The alerts were classified as “pertinent” or “non pertinent” i.e., whether or not they were associated with an action or intervention by the clinic.

## 3. Experimental Protocol

The MyPredi™ solution (i.e., a tablet and connected sensors) [7,8] was used to collect the patient’s physiological data, including blood pressure, heart rate, weight, oxygen saturation, capillary blood glucose, and temperature three times per day (morning, noon, and night). A number of physiological measurements were taken by the pedometer for physical activity and sleep. The patient wore the pedometer day and night, and the data (physical activity and sleep) were automatically sent to the MyPredi™ platform.

Additional information on geriatric risks and disorders was collected on a daily basis by way of questionnaires completed on the tablet [7,8]. These questionnaires addressed falls, constipation, dehydration, confusion, iatrogenesis, malnutrition, heart failure, hypertension, diabetes, confusion, infections, and bedsores. A therapy-related questionnaire was also completed by the caregivers in conjunction with the patient during the patient′s stay. Table 1 illustrates the geriatric risks and disorders that were monitored during the first experiment [8] and during the second phase with the addition of the “confusion” risk by the use of the CAM questionnaire, and the “neuropsychiatric disorders” by the use of the NPI questionnaire. We use the same detailed questionnaires embedded within the MyPredi™ platform [8] and used for monitoring the geriatric risks studied.

## 4. The Remote Monitoring Platform

The MyPredi™ platform is a generic platform with an original architecture and proven capabilities to refer patients with chronic pathologies who require long-term management [7].

This remote monitoring platform is based on non-intrusive medical sensors, allowing the collection of capillary blood glucose, blood pressure, heart rate, arterial oxygen saturation (SaO2), temperature and body weight. These sensors communicate via Bluetooth, enabling real-time feedback of physiological information on the patient’s health status. The platform also includes a touch tablet, which communicates via Wi-Fi with a box, or via 3G/4G, enabling interaction with the patient and providing nutritional-hygienic and therapeutic education. The MyPredi™ system includes a server that hosts patient data and a secure Internet portal (website), allowing the patient and the various healthcare professionals to connect.

MyPredi™ is based on an “intelligent” system in the form of an inference engine and a medical ontology, enabling personalized data analysis, which is specific to each patient, in real time or delayed mode, with, ultimately, the generation of “alerts”. The MyPredi™ platform generates “indicators of deterioration in the patient’s health status,” known as “alerts,” in relation to a decompensation of chronic pathologies. This reasoning is based on an inference engine whose rules are created by medical experts (here, cardiologists, internists, geriatricians and diabetologists,). The medical knowledge is derived from evidence-based medicine [7,8].

## 5. Parameters Evaluated and Statistical Analyses

We used RStudio software and R code (Boston, MA, USA).

We calculated sensitivity, specificity, and positive and negative predictive value for alerts issued for the geriatric risk. Survival analyses were estimated using the Kaplan-Meier method. For the comparison of living and deceased elderly patients, we used the Student’s *t*-test and the Wilcoxon test.

We used the training data for the logistic regression modeling and we used the test data to calculate accuracy and error. We used the cross validation and the polynomial kernel in the SVM modeling with the training data. Next, an effort was made to compute the accuracy and error measures obtained on the test data with the SVM model. The performance of the prediction is measured from the accuracy ie ; the precision of the model calculated from the confusion matrix. Accuracy = (true positive + true negative)/*n* with *n* = true positive + true negative + false positive + false negative. We managed to find a model with good precision.

## 6. Administrative Requirements

Written and signed consent was required for the inclusion of elderly patients. The experiment was approved by the Ethics Committee of the University Hospitals of Strasbourg and by the French National Commission for Information Technology and Civil Liberties (CNIL). The experiment is registered under the number RNI 2020—HUS No. 7792.

## 7. Results

### Characteristics of Patients

A total of 71 patients were hospitalized in the internal medicine unit between 14 December 2020 and 25 February 2021. Of these, 30 elderly patients affected by COVID-19 disease were monitored remotely during their hospitalization, while 41 patients did not meet the eligibility requirements (eight patients were monitored by the telemedicine solution for less than 48 h, 15 patients were under 65 years of age and nineteen were in palliative care on admission or died within 48 h of admission). The mean age of the patients was 85.9 years with a standard deviation of 6.4 years. The median age was 86.3 years. There were 18 (60%) male patients and 12 female patients: a male/female ratio of 1.5 to 1. The patients used the telemedicine solution for an average of 27.3 days. Among the 30 patients, 15 (50%) had a history of heart deficiency, 16 (53.3%) had a history of hypertension, five (16.7%) had a history of asthma/COPD, 10 (33.3%) had a history of diabetes, and four (13.3%) had a history of solid tumors. See Table 2 for more information on the accompanying syndromes. The average number of drug treatments at the time of admission was eight, with a standard deviation of 4.1. See Table 2 for more information on the treatments. The mean Charlson score was 6 (5–12) with a standard deviation of 1.2. The average length of stay was 14.3 days (4–55).

All of the patients had a positive result for SARS-CoV-2 confirmed by reverse transcriptase PCR (RT-PCR) tests on nasopharyngeal swabs (thereafter referred to as COVID-19 patients).

Of the 30 COVID-19 patients, 21 (70%) lived at home and nine (30%) lived in nursing homes. After hospitalization, 12 (40%) patients returned to their homes, six (20%) went to nursing homes, and one patient was transferred to a rehabilitation center for further treatment. 11 (36.7%) died during the experiment.

## 8. Data from the Sensors/Questionnaires

The MyPredi™ remote monitoring solution collected a total of 140,260 measurements while monitoring the geriatric syndromes of the entire patient group. On average, 4675 measurements were recorded per patient for geriatric disorders and risks. On average, 319 measurements were recorded per patient per day. Our results, in the comparison between the two phases, reveal that oxygen saturation is lower, a significant hyperglycemia was noticed, lower physical activity was measured by the pedometer, and a stool frequency significantly reduced in elderly subjects affected by COVID-19. See Table 3 for more general and collected data from sensors/questionnaires; we compare these data of these two phases.

We also present the results of the comparison of elderly patients who survived COVID-19 infection to the series of elderly patients who died from COVID-19 infection. In these data, our results show significantly lower systolic blood pressure, reduced stool rate, higher heart rate, reduced oxygen saturation and significant reduction in daily physical activity in elderly subjects who died from COVID-19-related infection. See Table 4 for detailed results.

## 9. Number of Alerts for Geriatric Syndromes/Chronic Diseases

The telemedicine solution emitted a total of 1245 alerts while monitoring the geriatric syndromes of the entire patient group. For each geriatric risk/disorder, an average of 42 alerts were emitted per patient, with 7 of these alerts classified as “low”, 12 classified as “medium”, and 23 classified as “critical”.

Table 5 illustrates the criticality of the alerts for each of the geriatric syndromes. No alerts were emitted for the “bedsore” risks. In the total population, a mean number of 3 alerts per day per patient was observed.

In our additional analyzes, 29 alerts (2.3%) were emitted for the “kidney failure” risk, with an average of 2.6 alerts per patient and a standard deviation of 1.9. Less frequently, 11 alerts (0.9%) were emitted for the “decrease or increase in heart rate” risk, with an average of 1.8 alerts per patient and a standard deviation of 1.2; 18 alerts (1.4%) were emitted for the “insufficient physical activity” risk, with an average of three alerts per patient, with a standard deviation of 1.7.

In total, 156 alerts were issued for “hallucinations,” an average of 52 per patient. For “hallucinations,” we obtained a mean score of 1.8 for the entire study group and an average of 6.2 per patient. In total, 1048 alerts were issued for “agitation/aggressiveness,” and an average of 52.4 per patient. For “agitation/aggressiveness,” we obtained a mean score of 1.89 for the entire study group and an average of two per patient. In total, 1572 alerts were issued for “anxiety,” and an average of 92.5 per patient. For “anxiety,” we obtained a mean score of 1.7 for the entire study group and an average of 3.6 per patient. In total, 649 alerts were issued for “apathy/indifference,” and an average of 54.1 per patient. We obtained a mean score of 1.86 for the entire study group and an average of 4.7 per patient. In total, 124 alerts were issued for “delusional ideas” and an average of 41.3 per patient. We obtained a mean score of two for the entire study group and an average of 1.3 per patient.

## 10. Clinical Relevance of Alerts

Table 6 and Table 7 illustrates the clinical relevance of the alerts in terms of Se, Spe, PPV, and NPV for the evaluated criteria. Note the sensitivity of 100% for the alerts of all the evaluated geriatric risks and the high negative predictive value.

**Figure 1:**
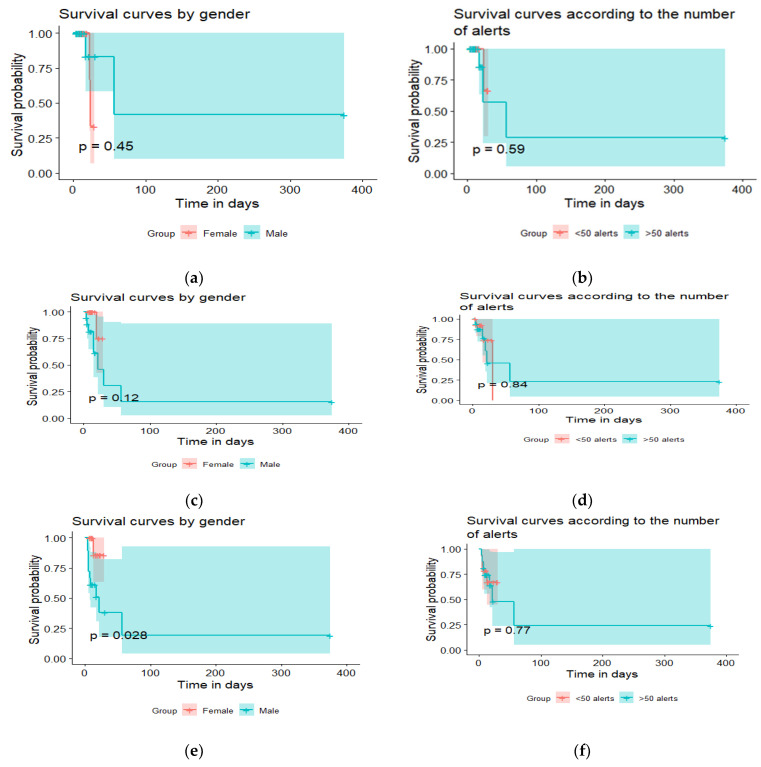
Survival analysis. (**a**) Survival curves by gender following cardiac decompensation. (**b**) Survival curves by number of alerts following cardiac decompensation. (**c**) Survival curves by gender following alteration of general health. (**d**) Survival curves by number of alerts following alteration of general health. (**e**) Survival curves by gender following death. (**f**) Survival curves by number of alerts following death.

Survival analyses (Figure 1) showed that gender played no role in the length of the hospital stay, regardless of the reason for the hospitalization (decompensated heart failure (*p* = 0.45), deterioration of general condition (*p* = 0.12), but significant for death (*p* = 0.028)). The analyses revealed that the length of the hospital stay was not affected by the number of alerts (decompensated heart failure (*p* = 0.59), deterioration of general condition (*p* = 0.84), and for death (*p*-value = 0.77)).

## 11. Multivariate Analysis and Alert Prediction

The goal of the study is to determine through a one-factor analysis of variance (ANOVA test) whether we have the same level of criticality regardless of patient age. It is concluded that the criticality level varies with age (*p*-value = 0.0003369).

We now want to determine through a one-factor analysis of variance (ANOVA test) if we have the same level of criticality regardless of length of hospitalization. It has been stated that the level of criticality varies depending on the length of hospitalization (*p*-value = 3.803 × 10^−10^.

### 11.1. Logistic Regression

The logistic regression model is used to study the relationship between a qualitative variable of binary interest Y and one or more explanatory variables that may be quantitative or qualitative.

Let Y equal the binary qualitative variable representing the criticality level of the alerts. X1 represents the geriatric risks, X2, the sex, X3, the age, X4, the treatment, X5, the length of a hospital stay, and X6 the types of pathology.

The best model obtained is the following with two significant variables (treatment and pathology) at the threshold of 10%: Y (criticality level) = X1 (geriatric risk) + X2 (treatment) + X3 (age) + X4 (pathology). Length of hospital stay is not a significant variable in the chosen model. As we have noted in the ANOVA, the length of a hospital stay does not have a significant impact on the criticality level of the alerts. Age is retained in the model but is not significant.

. Accuracy
  88.7% of alerts are well classified.
. error
  The model does not correctly predict critical or urgent alerts in 11.3% of cases.
. Sensitivity
  Of the predicted critical or urgent alerts, 98.7% are observed in the sample.
. Specificity
  Of the predicted low or moderate alerts, 75.4% are observed in the sample.
. Positive predictive value
  Of the critical or urgent alerts observed, 84.1% are predicted by the model.
. Negative predictive value
  Of the low or moderate alerts observed, 97.8% are predicted by the model.

### 11.2. SVM Modeling

Support Vector Machine (SVM) is a family of algorithms dedicated to regression and supervised classification problems. The approach presented here is the one that is used in the case of supervised classification with a variable to explain binary Y. We den. X= (X1,...,Xp), the vector of explanatory variables. So we’ll apply a machine learning algorithm on our dataset that will learn from the data and that will then be able to determine the nature of the alert (or not) from all the variables in our dataset, but without having the prior response of the criticality level of the alert. We created a training sample containing 75% of our observations to estimate the models and a test sample for the rest of our observations. We have eight variables, and this makes sense because in order to have good precision, we use the reduction of the dimension and the. The dummification step for each qualitative variable in consists in creating a binary variable for each of its modalities.

**Figure 2:**
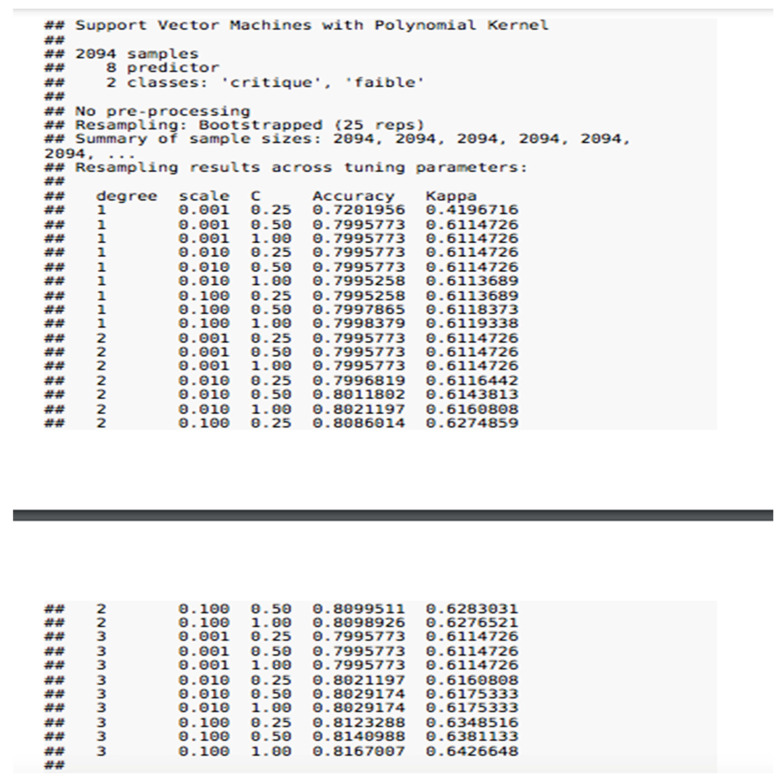
SVM model.

**Figure 3:**
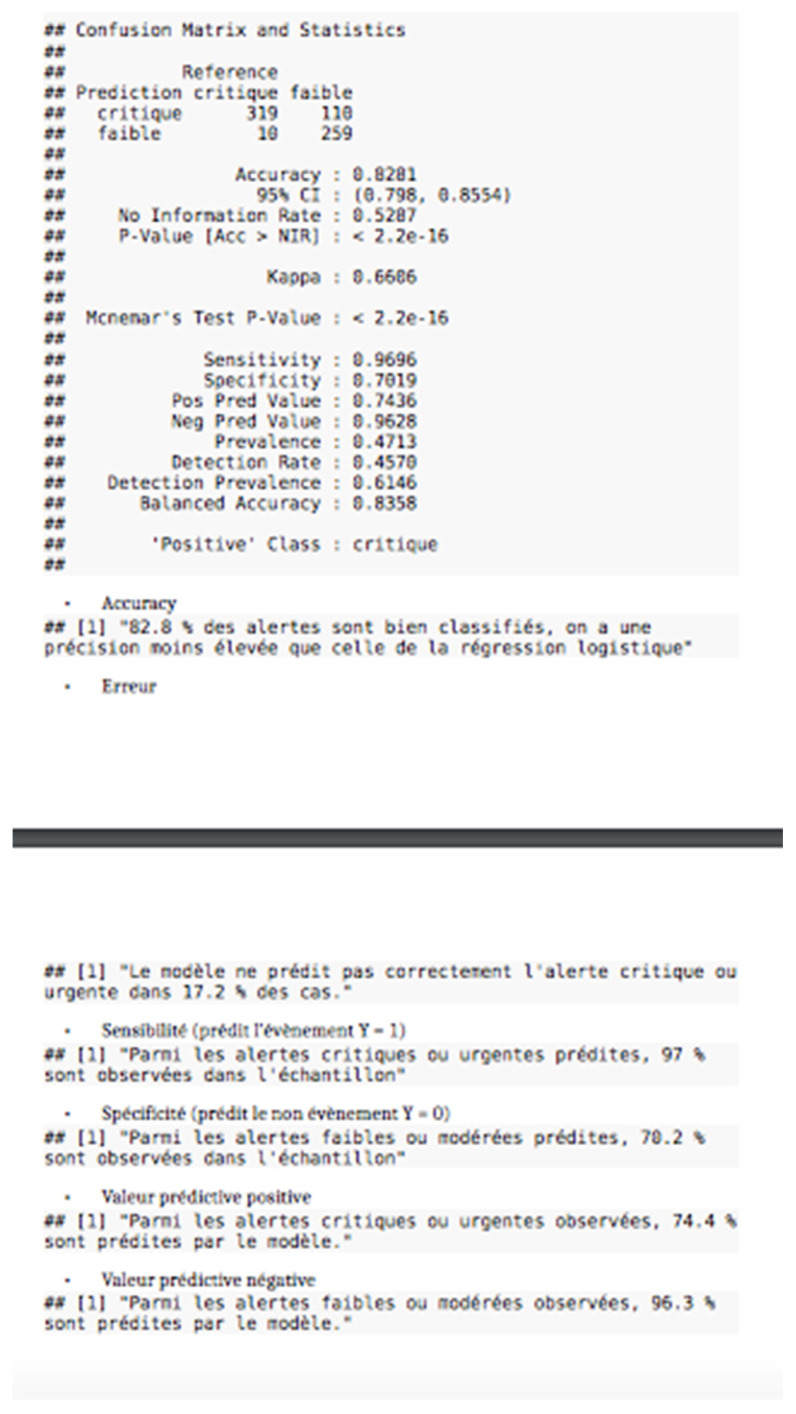
Confusion matrix.

In Figure 2, we can clearly see that we have eight explanatory variables with (8 predictor) marked in the image for the SVM. See Figure 3, the confusion matrix.

. Accuracy
  82.8% of alerts are well classified. We therefore have a lower accuracy rate than with logistic regression.
. error
  The model does not correctly predict critical or urgent alerts in 17.2% of cases.
. Sensitivity
  Of the predicted critical or urgent alerts, 97% are observed in the sample.
. Specificity
  Of the predicted low or moderate alerts, 70.2% are observed in the sample.
. Positive predictive value
  Of the critical or urgent alerts observed, 74.4% are predicted by the model.
. Negative predictive value
  Of the low or moderate alerts observed, 96.3% are predicted by the model.

## 12. Discussion

This is the second phase of the GER-e-TEC study, with new data concerning elderly subjects with infection linked to COVID-19. The first phase, which was published in the JCM journal [8], took place between September 2019 and November 2019 (before the advent of COVID-19), and corresponded to an experimental phase. This second phase corresponds to a phase of validation and confirmation of the technological choices made during the first phase. In addition, the novelty of this second phase lies in the telemonitoring of elderly patients hospitalized with COVID-19 infection, which in the scientific literature (PubMed, Google Scholar) has not been carried out so far in the case of remote monitoring of geriatric risks. In this study, the objective was to study the impact of COVID-19 on geriatric risks. This second phase brings innovative elements in the prediction of alerts, which had not been achieved during the first phase. To the best of our knowledge, taking into account the scientific literature from the main scientific search engines (PubMed, Google Scholar), our telemonitoring work is innovative in the prevention of decompensations of geriatric risks in a population of elderly subjects affected by infection linked to COVID-19 infection.

COVID-19 infection affected the experiment we conducted, since during the second phase, eleven elderly people with COVID-19 died, whereas during the first phase, there were no deaths of elderly NON-COVID patients.

Beyond hemodynamic parameters such as blood pressure, oxygen saturation being significantly lower in elderly COVID patients and especially in elderly people with COVID who died, and hyperglycemia being significantly higher in elderly people with COVID who died, we noted that physical activity measured by the pedometer, the risk of prolonged bed rest (assessed by the use of a questionnaire) and the frequency of stools were significantly lower in elderly people with COVID-19 infection and mainly in elderly people with COVID who died.

Moreover, the survival analyses of patients hospitalized for “decompensated heart failure”, “alteration of general condition”, and “death” produced some interesting results. Survival analyses showed that gender played no role in the length of the hospital stay, regardless of the reason for the hospitalization (decompensated heart failure (*p* = 0.45), deterioration of general condition (*p* = 0.12), but significant for death due to COVID-19 disease (*p* = 0.028)).

Our results also showed that the MyPredi™ remote monitoring platform is effective at automatically and non-intrusively generating alerts in the event of increased geriatric risks, in particular those associated with pain, heart rate, bed rest, confusion, hypertension, diabetes, fever and decompensated heart failure in COVID-19 older patients. In fact, the system is most adept at detecting these risks, with sensitivity and positive predictive values of 100%. The results concerning the predictive nature of alerts are satisfactory regardless of the method used.

In the literature, very few studies have focused on the telemonitoring of the geriatric population affected by COVID-19 infection. During COVID-19, telemedicine has been used to triage, treat, and coordinate the provision of care to patients to improve health care access, reduce disease transmission, and optimize resource allocation [9,10,11,12,13]. O’Keefe et al., established a virtual clinic for the care of patients in home isolation with COVID-19, known as the ‘Virtual Outpatient Management Clinic’ (VOMC), and patients were followed for symptom management with regular telephone calls by registered nurses (RNs) and APPs until improvement or hospitalization [14]. Telemedicine has emerged as a viable tool for the delivery of healthcare in lieu of in-person patient contact. The variable and occasionally rapid course of clinical disease raises safety concerns in using telemedicine in the clinical management of acute infection with the novel coronavirus [15]. In Australia, Hutchings et al., developed a virtual health care program or community management of patients with COVID-19 [16]. It was an observational cohort study that included patients with COVID-19 with an established virtual health care program capable of monitoring patients remotely. Skin temperature, pulse rate, and blood oxygen saturation were remotely monitored. A total of 162 of 173 (93.6%) patients with COVID-19 (median age 38 years, range 11–79 years), who were diagnosed locally, were enrolled in the virtual health care program. Video consultations (*n* = 1902, 66.3%) comprised most of the patient contacts, and 132 (81.5%) patients were monitored remotely.

Motta LP et al., developed an emergency system for monitoring pulse oximetry, peak expiratory flow, and body temperature of patients with COVID-19 at home [17]. Their system consisted of the home-based patient unit, which is set up around the patient and the hospital unit, which enables the medical staff to telemonitor the patient’s condition and to send medical recommendations when required. The home unit allows the data transmission from the patient to the hospital, which is performed using a cell phone application [17]. Silven et al., developed the “COVID Box” telemonitoring care pathway which was implemented as standard care for patients with COVID-19. The COVID Box was a program developed by the Leiden University Medical Center to monitor patients with confirmed or suspected COVID-19 who have an increased risk of severe illness. This COVID Box contained a pulse oximeter, blood pressure monitor, thermometer, and information folders. Patients were selected for telemonitoring when visiting the emergency care department for (suspected) COVID-19 (after referral by their general practitioner), or after admission to the COVID-19 department. 55 patients were monitored at home using the COVID Box Between 1 March and 15 June 2020 [18]. Telemonitoring offers the opportunity to carefully monitor patients with a confirmed or suspected case of COVID-19 from home and allows for the timely identification of worsening symptoms [18].

As such, telemonitoring may enable early identification of the deterioration of symptoms, and allows for appropriate treatments for each patient with COVID-19.

Thanks to the MyPredi™ remote monitoring platform, patients benefit from personalized medical follow-up that allows caregivers to prevent acute deteriorations in their condition. Our project arose from the need to improve care provided in nursing homes by combining digital transformation, the needs of the elderly, and the five P’s of medicine (predictive, preventive, personalized, participatory, and purpose-driven).

The main limitation of our work lies in the number of alerts issued by our remote monitoring system, like the number of alerts issued for neuro-psychobehavioral disorders evaluated by the NPI questionnaire. It will be necessary to make adjustments to ensure the system does not become overloaded.

In addition, fall risk has not been specifically studied. This remains a real problem for the elderly and, in particular, the institutionalized elderly. The risk of falling is one of the most significant risks among the elderly and leads to increased mortality in patients with illnesses. Our remote monitoring solution will soon be enhanced by the use of connected insoles which will allow us to better understand the risk of falling by studying the posture and walking of patients.

## 13. Conclusions

The “GERETEC” remote monitoring project will soon be implemented in the “Les Opalines” group of retirement homes to improve the quality of life of elderly residents and prevent the deterioration of geriatric syndromes (and consequently, visits to emergency rooms), as well as at the homes of patients in the city of Angers in the Pays de la Loire region of France.

## Figures and Tables

**Table 1:** Remote monitoring of geriatric risks in the GER-e-TEC study [8].

Geriatric Risk	Connected Sensors/Questionnaires	Frequency
Hemodynamic data (hypertension/hypotension–tachycardia/bradycardia–oxygen desaturation/infections)	Sphygmomanometer–pulse oximeter–thermometer	Three times per day
Heart failure	Questionnaire	Daily
Constipation	Questionnaire	Twice a day
Risk of bed rest	Questionnaire Pedometer	Daily Daily
Pain	Questionnaire	Daily
Dehydration	Questionnaire Biological sensors (natremia–kaliemia–creatinine)	Daily Twice a week
Sleep quality	Pedometer	Day and night
Physical activity	Pedometer Questionnaire	Daily Daily
Diabetes	Glucometer	Three times per day
Iatrogenism	Questionnaire	On admission and once during hospitalization
Malnutrition	Balance Biological sensor (albumin)	Twice a week Once during hospitalization
Confusion	Questionnaire (CAM *)	Daily
Neuropsychiatric disorders	Questionnaire (NPI *)	Twice a day

CAM *: Confusion Assessment Method. NPI *: NeuroPsychiatric Inventory.

**Table 2:** Characteristics of the study population (*n* = 30).

Medical Characteristics (*n*, %)	
Medical History	
Heart deficiency	15 (50%)
Arterial hypertension	16 (53.3%)
Atrial fibrillation	8 (26.7%)
Coronary syndrome	9 (30%)
Pacemaker	4 (13.3%)
Obliterating arteriopathy of the lower limbs	1 (3.3%)
Sleep apnea syndrome	1 (3.3%)
Phlebitis/pulmonary embolism	5 (16.7%)
Dyslipidemia	9 (30%)
Diabetes	10 (33.3%)
Stroke	7 (23.3%)
Chronic renal deficiency	4 (13.3%)
COPD *	5 (16.7%)
Solids neoplasms	4 (13.3%)
Cirrhosis	1 (3.3%)
Peptic ulcer	2 (6.7%)
Hypothyroidism	4 (13.3%)
Connectivities	3 (10%)
Cognitive disorder	15 (50%)
Treatment	
Beta blockers	12 (40%)
ACE inhibitors, Sartan	11 (36.7%)
Diuretics	13 (43.3%)
Calcium channel blockers	9 (30%)
Anticoagulants	8 (26.7%)
Antiplatelet agents	9 (30%)
Statins	9 (30%)
Oral antidiabetics	5 (16.7%)
Insulin therapy	2 (6.7%)
Benzodiazepines	14 (46.7%)
Antipsychotics	5 (16.7%)
Antidepressant	5 (16.7%)
Proton pump inhibitors	13 (43.3%)
L-Thyroxin	4 (13.3%)
Antiarrhythmics	4 (13.3%)
Symptoms at onset of illness COVID-19	
Fatigue	12 (40%)
Confusion	6 (20%)
Dehydratation	7 (23.3%)
Dyspnoea	22 (73.3%)
Fever	12 (40%)
Cough	11 (36.7%)
Diarrhoae	4 (13.3%)
Acute heart failure	3 (10%)
Pulmonary embolism/phlebitis	3 (10%)
Arterial thrombosis	2 (6.7%)
Asymptomatic	1 (3.3%)
Total lung involvement Chest CT Findings in Coronavirus Disease-19 (COVID-19)	
Minimal (<25%)	17 (56.7%)
Moderate (25–50%)	7 (23.3%)
Severe to critical (>=75%)	6 (20%)

COPD *: Chronic obstructive pulmonary disease.

**Table 3:** General and collected data from sensors and questionnaires during the two phases of the Ger-e-Tec study.

General Data	First Phase GER-e-TEC (*n* = 36) [8]	Second Phase GER-e-TEC COVID (*n* = 30)	*p*
Age	81.4 (±7.7)	85.9 (±6.4)	0.0053
Average use of the telemedicine solution	22.1	27.3	0.6575
Average number of drug treatments	8.5 (±4.2)	8 (±4.1)	0.6964
Charlson score	6.86	6	1
Average measurements recorded per patient for geriatric disorders	4476	4675	0.5408
Average measurements recorded per patient per day	226	319	<2.2 × 10^−16^
Data from Sensors and Questionnaires (Mean ± Standard Derivation)			*p*
Arterial pressure	105.70 mm Hg (±8.1 mm Hg)	105.72 mm Hg (±8.12 mm Hg)	0.5023/0.9986
Heart rate	77.6 bpm (±15 bpm)	80.4 bpm (±15.2 bpm)	7.831 × 10^−6^
Oxygen saturation	96.5% (±21)	94.2% (±4.1)	<2.2 × 10^−16^
Blood glucose level	124.3 mg/L (±86 mg/L)	163.2 mg/L (±86.7 mg/L)	4.196 × 10^−6^
Weight	75.1 kg (±23.1 kg)	68.5 kg (±14.4 kg)	0.9937
Temperature	36.7 °C (±0.6 °C)	36.5 °C (±0.9 °C)	3.162 × 10^−9^
Physical activity (median)	394 steps per day	212.5 steps per day	3.13 × 10^−7^
Daily activity index	13.9% (±14.1%)	10.6% (±11.3%)	0.01341
Amount of sleep	500.3 min per day (±206 min)	492.5 min per day (±135.7 min	0.188
Amount of light sleep	139.8 min per day (±144.4 min)	124.1 min per day (±126.1 min)	0.9495
Amount of deep sleep	358.8 min per day (±159.2 min)	368.4 min per day (±137.8 min)	0.02305
Stool frequency	0.6 stools per day (±0.6)	0.4 stools per day (±0.6)	<2.2 × 10^−16^
VAS pain score	1.2 (±0.3)	0.4 (±0.5)	<2.2 × 10^−16^
VRS pain score	0.6 (±0.2)	0.1 (±1.2)	<2.2 × 10^−16^
Algoplus	7.5 (±2.3)	0.9 (±1.7)	0.5
Albumin level	35.2 g/L (4.1 g/L)	36.2 g/L (3.8 g/L)	0.074
Natremia	136.2 mmol/L (±3.6 mmol/L)	140.3 mmol/L (±6.2 mmol/L)	1.941 × 10^−12^
Kalemia	4.2 mEq/L (±0.6 mEq/L)	4.1 mEq/L (±0.6 mEq/L)	0.01169
Creatinine level	87.3 µmol/L (±30.2 µmol/L)	80.8 µmol/L (±26.3 µmol/L)	0.9487
INR *	2.5 (±1.4)	3.4 (±2.4)	0.6226
Vitamin D	-	29 ng/mL (±33.6)	-

* Based on only two International Normalized Ratio (INR) measurements.

**Table 4:** Elderly patients COVID-19 alive VS elderly patients COVID-19 deceased.

General Data	Elderly Patients COVID-19 Alive (*n* = 19)	Elderly Patients COVID-19 Deceased (*n* = 11)	*p*
Age	85.1 (±5.2)	87.4 (±8.1)	0.9074
Average use of the telemedicine solution	35.1 (±82.6)	13.7 (±15.5)	0.8569
Average number of drug treatments	7.9 (±4.6)	8.1 (±3)	0.5406
Charlson score	5.7 (±1.1)	6.5 (±1.4)	0.925
Average measurements recorded per patient for geriatric disorders	5015	3876	0.03176
Average measurements recorded per patient per day	322	302	0.9979
Albumin level	37 (±4.3)	34.6 (±2.6)	0.9857
Natremia	139.2 mmol/L (±5.8 mmol/L)	142.2 mmol/L (±7 mmol/L)	0.002776
Kalemia	4 mEq/L (±0.5 mEq/L)	4.2 mEq/L (±0.7 mEq/L)	0.8188
Creatinine level	80.5 µmol/L (±25.8 µmol/L)	81.6 µmol/L (±27.9 µmol/L)	0.5608
INR	2.9 (±1.8)	4.1 (±3.2)	0.6537
Stool frequency	0.5 stools per day (±0.6)	0.2 stools per day (±0.5)	1.047 × 10^−6^
Arterial pressure	107.72 mm Hg (±6.13 mm Hg)	101.71 mm Hg (±9.12 mm Hg)	6.607 × 10^−5^/0.08317
Heart rate	79.9 bpm (±14.5 bpm)	82.4 bpm (±16.8 bpm)	0.02348
Oxygen saturation	95.3 % (±2.6)	92.2 % (±5.4)	<2.2 × 10^−16^
Blood glucose level	151.3 mg/L (±92.6 mg/L)	210.8 mg/L (±60.6 mg/L)	2.165 × 10^−14^
Weight	68 kg (±17.3 kg)	70.2 kg (±10.3 kg)	0.05735
Temperature	36.4 °C (±0.8 °C)	36.4 °C (±0.9 °C)	0.9151
Physical activity	840.1 steps per day (±1222.7)	170.2 steps per day (±328.7)	7.912 × 10^−13^
Daily activity index	12.9% (±13%)	4.3% (±3.7%)	8.58 × 10^−5^
VAS pain score	0.3 (±1)	0.5 (±1.7)	0.71
VRS pain score	0.1 (±0.4)	0.2 (±0.6)	0.5218
Amount of sleep	484 min per day (±123.1 min)	497.3 min per day (±160.1 min)	0.6248
Amount of light sleep	140.7 min per day (±112.5 min)	90 min per day (±155.3 min)	1
Amount of deep sleep	343.3 min per day (±140.4 min)	407.3 min per day (±136.2 min)	3.749 × 10^−7^
Vitamin D	33.4 ng/mL (±38.9)	19.7 ng/mL (±15.2)	0.8822

**Table 5:** Total alerts emitted per risk group and geriatric risk (“low”, “medium”, and “critical”).

Geriatric Syndromes	Alerts Total	Low Alerts	Moderate Alerts	Critical Alerts
Bed rest	159	159 (100%)	0	0
Confusion	9	0	0	9 (100%)
Constipation	28	0	28 (100%)	0
Tachy–bradycardia	11	0	0	11 (100%)
Malnutrition	18	0	17 (94.6%)	1 (5.5%)
Pain	33	0	33 (100%)	0
Hyperthermia	11	0	11 (100%)	0
Hypo- and hyperkalemia	29	0	29 (100%)	0
Hypo- and hypernatremia	18	0	18 (100%)	0
Hypo- and hypertension	238	0	0	238 (100%)
Iatrogenesis	244	44 (18%)	58 (23.8%)	142 (58.2%)
Heart failure	413	0	126 (30.5%)	287 (69.5%)
Hypertension	238	0	0	238 (100%)
Dehydration	34	0	34 (100%)	0
Diabetes	142	69 (48.6%)	38 (26.8%)	35 (24.6%)

**Table 6:** Sensitivity, specificity, and positive and negative predictive values for alerts from the MyPredi™ remote monitoring platform.

	Decompensated Heart Failure	Pain	Dehydration	Brady-and Tachycardia	Constipation	Bed Rest	Malnutrition	Iatrogenia	Confusion
Sensitivity	100%	100%	100%	100%	100%	100%	100%	100%	100%
Specificity	-	-	50%	50%	50%	-	49%	49%	-
Positive predictive value	100%	100%	58%	73%	34%	100%	29%	42%	100%
Negative predictive value	-	-	100%	100%	100%	-	100%	100%	-

**Table 7:** Sensitivity, specificity, and positive and negative predictive values for alerts from the MyPredi™ remote monitoring platform.

	Fever	Hypo-and Hyperkalemia	Hypo-and Hypernatremia	Diabetes	Hypertension	Agitation/Aggression	Hallucinations	Anxiety	Apathy/Indifference	Delusion
Sensitivity	100%	100%	100%	100%	100%	100%	100%	100%	100%	100%
Specificity	-	49%	50%	-	-	-	-	-	-	-
Positive predictive value	100%	43%	35%	100%	100%	100%	100%	100%	100%	100%
Negative predictive value	-	100%	100%	-	-	-	-	-	-	-

## Data Availability

The datasets used and/or analyzed during the current study are available from the corresponding author on reasonable request.
